# Leaving Academia: Dual-Career Relationships and Partners’ Attrition from Academic Careers

**DOI:** 10.1177/26884844251366373

**Published:** 2025-08-11

**Authors:** Jill A. Fisher, Yu Tao, Margaret Waltz, Torin Monahan

**Affiliations:** ^1^ Department of Social Medicine and Center for Bioethics, University of North Carolina at Chapel Hill, Chapel Hill, North Carolina, USA.; ^2^ School of Humanities, Arts and Social Sciences, Stevens Institute of Technology, Hoboken, New Jersey, USA.; ^3^ Department of Communication, University of North Carolina at Chapel Hill, Chapel Hill, North Carolina, USA.

**Keywords:** academic couples, two-body problem, partner hiring, dual-career, leaky pipeline, survey

## Abstract

**Background:**

More than one third of academics are coupled with another academic, with more women being in such dual-career relationships. Little is known about how these couples’ experiences affect their attrition from or persistence in academia.

**Methods:**

We analyzed survey data of academics at 100 U.S. colleges and universities to answer two research questions: (1) Among all academic partners, who are the most likely to abandon their desired academic careers in terms of their gender, race, and field? (2) What effects does leaving academia have on those partners’ career outcomes?

**Results:**

We found that 22% of aspiring academics in academic relationships leave that career pathway. One third leave for personal reasons, including to prioritize their partner’s career. When partners leave academia for personal reasons, they are less likely to be employed in any job and, when employed, are paid less than their counterparts who leave academia for professional reasons. Among our results, we found notable gender differences. Compared with men, women in medicine were more likely to leave academia for personal reasons. Moreover, the earnings of women who leave academia due to personal reasons are the most negatively impacted.

**Conclusions:**

These trends indicate that the choices made by dual-career couples in response to the academic job market and to universities’ policies for partner hiring have substantial effects on the demographic makeup of academic research and scholarship. By supporting the needs of academic couples, universities have the opportunity to make their own institutions more diverse and to patch a hole in the leaky pipeline.

## Introduction

There are many obstacles to a long academic or scientific career. Research on the recruitment of students to enter science, technology, engineering, and mathematics (STEM) fields and medicine has historically utilized the metaphor of the pipeline to characterize the flow of those students into careers.^1,2^ The “leaky” pipeline metaphor, in particular, has been used to draw attention to the attrition of scientists, especially women and underrepresented racialized minorities, at all stages of scientific careers. More recently, scholars have advocated for the use of the term “pathway” to grant more agency to those pursuing such careers as they choose their own way in educational and professional settings.^3,4^ The benefits of a pathway framing are many, including its focus on what interventions can improve persistence in STEM fields and academic medicine.^5,6^ However, the pathway metaphor can also absolve institutions of the responsibility they bear in pushing some academic researchers from the careers they have chosen.

One important but underappreciated obstacle to some academics’ persistence in their chosen careers is whether they are part of a dual-career couple. Previous research has shown that about one third of all academics have a partner who is also an academic.^7^ Having an academic partner is common for all racial groups, but a slightly higher percentage of White compared with Asian, Black, Latinx, and other minoritized scholars are in these relationships (36% vs. 31%, respectively).^7,8^ Women are more likely than men to have an academic partner, and this is especially the case for women in STEM fields, with 59% being in such relationships.^9^ Women physicians are also more likely to be coupled with another physician than are their men counterparts.^10^ Thus, dual-career issues affect women more than men, especially those in STEM and medicine. Much of the literature has focused on gender and dual-career relationships, providing substantial evidence that women make greater career sacrifices for their relationships,^11–13^ and such sacrifices may include attrition from academic careers.^14^ Regarding women of color in particular, their career sacrifices have been shown to bring them to positions that are a poor fit, affecting their job satisfaction, experience of inclusion, and retention in the academy.^15^

Universities have become more aware of the need to accommodate dual-career academic couples as part of recruitment and retention of faculty,^16^ and their “partner hiring” programs may be especially critical for increasing the representation of women in STEM and medicine. For example, one study found that partner hiring helped increase the number of women in senior ranks at medical schools.^17^ However, studies also indicate that many universities still make no effort to facilitate the employment of a candidate’s partner at their institution or in the community and may even discriminate against candidates with dual-career partners.^18–20^ LGBTQ+ couples can face even greater bias in recruitment, especially at religious or conservative academic institutions.^21,22^ By not accommodating the needs of dual-career academics, institutions risk losing top candidates during job searches or failing to retain exceptional faculty.^16,17,23^ Even when “accommodations” are made, academic partners can be subjected to the stigma of being labeled as a “trailing spouse” or forced into positions that are a poor fit or fail to match their professional goals.^15,24^ As a result, dual-career issues may be an overlooked contributor to the leaky pipeline, pushing out individuals who would otherwise choose an academic research career.^14,25–28^

While there is a small—and mostly qualitative—literature on the impact of university hiring practices on academic couples’ careers,^29,30^ there has been little quantitative research on the experiences of dual-career academics and how their relationship priorities may affect their careers. We conducted a national survey on perceptions and experiences of university partner hiring. This research also queried how academic couples’ experiences on the job market affect their career pathways and outcomes. We analyze data here to answer two research questions: (1) Among all academic partners, who are the most likely to abandon their desired academic careers in terms of their gender, race, and field? (2) What effects does leaving academia have on those partners’ career outcomes? This investigation provides insight into the career consequences for couples who intended to pursue academic careers together when one member of the couple ultimately left academia.

## Materials and Methods

### Data source

Our sample consisted of academics at 100 colleges and universities in the United States. We sampled diverse institutions across 33 states. To select institutions, we used stratified random sampling, ensuring representation of institution types based on their Carnegie Classification, region, and institutional control (*i.e.,* public or private). We then identified potential survey respondents at each institution through directory searches. We emailed all faculty and postdoctoral fellows, as well as administrative and research staff with advanced degrees that would typically qualify someone for a faculty position, to invite them to participate in an online Qualtrics survey. The survey was administered from October 26, 2022, to January 24, 2023. Our final sample for the overall project included 16,292 respondents, with a response rate of 22.5%. More details about the institutions included in the sample (Supplementary Table S1) can be found in the Supplementary Tables.

For the research reported here, we used a sample of 4,425 respondents who had an academic partner or a partner who had intended to be an academic. These partners included people who currently held, had retired from, previously applied for, or had intended to (but did not) apply for an academic job. We report on survey questions about attrition, the reasons that partners stopped looking for an academic job, and those partners’ current employment status and salary.

### Measures

Survey respondents whose partners were no longer pursuing an academic career were asked the reason their partner stopped looking. They were given a list of potential reasons to choose from as well as the opportunity to select “Other” and write in a response. Respondents were allowed to select all that applied, which included the following reasons: “[They] obtained a non-faculty position at a college or university”; “Employment opportunities outside of academia [were] more appealing”; “[They were] disappointed with the academic job market” or “[The] academic job market [was] too competitive to pursue”; “[They were] disappointed with academia more generally” or “[They] dislike the academic environment”; “[They] prioritized my opportunities for an academic career instead”; and “[They] prioritized home or family responsibilities instead.” For the purposes of analysis, we created a new binary variable which categorized these responses as “personal reasons” or “professional reasons.” If the respondent chose either “prioritized my opportunities for an academic career instead” or “prioritized home or family responsibilities instead,” they were categorized as having left academia for personal reasons. All others were categorized as having left for only professional reasons, which include employment opportunities (*i.e.,* more appealing nonacademic jobs) and dissatisfaction with academia related to the job market or university culture. If they selected both personal and professional reasons, we coded them as personal reasons in this analysis.

### Analytic approach

For the first research question on attrition, the dependent variable was categorical with three values: 1 = partners who stayed in academia (*i.e.,* current or retired academic partners), 2 = partners who left academia for professional reasons, 3 = partners who left academia for personal reasons. The latter two groups had previously held an academic job, had applied for such jobs but stopped their academic job search, or intended to apply for an academic job but did not; however, they differed in the reasons why they left academia. We used multinomial logistic regressions to test the effects of independent variables on attrition. To answer the second research question on career outcomes, we used the dependent variables of current employment status (full-time employment or not) and current salary, if employed and reported an income. We used logit and ordinary least squares regressions to test the effects of independent variables on employment and earnings, respectively.

The key independent variables for attrition (RQ1) include partners’ gender, race, and field. In addition to the main effects, we examined the three interaction effects between partners’ gender, race, and field, and for significant interaction effects, we also tested marginal effects. For gender, we included women, men, and other genders, which include those who identified as not women or men and those who did not indicate their gender. Because the other gender group included partners for whom we did not know their gender and was, thus, not a coherent demographic group, we focus only on results for men and women in our findings. We included in our analysis 6 racial/ethnic groups: non-Hispanic White, non-Hispanic Asian, non-Hispanic Black, Hispanic, Not Reported, and Others. The “Not Reported” group included people (from various racial/ethnic groups) who preferred not to provide that information, and the “Others” group combined smaller groups identified as American Indian, Middle Eastern and North African, multiracial, and others. Because the latter two groups were either a non-group (*i.e.,* no reported race or ethnicity data) or a heterogenous group created to combine smaller groups, we report findings only for the first four racial groups if the results were statistically significant. We included four broad fields in this research: (1) arts, humanities, and social sciences; (2) natural sciences, engineering, and mathematics (STEM hereafter); (3) medicine, nursing, and health sciences (medicine hereafter); and (4) professional fields, including business, education, law, architecture or planning, and journalism. The key independent variables for partners’ current career outcomes (RQ2) included attrition reason (personal or professional), gender, race, and field. We tested interaction effects between attrition reason and each of the three demographic variables (gender, race, and field), as well as marginal effects for significant interaction effects. Information regarding control variables can be found in Supplementary Tables S2 and S3.

## Results

### Descriptive findings

Our sample (4,425) of partners included more men than women (52.8% vs. 44.8%) and was predominantly non-Hispanic White (69.7%). The plurality of partners (42.8%) was in the arts, humanities, and social sciences, followed by 29.1% in STEM, 16.5% in medicine, and 11.6% in professional fields (Table 1). In terms of dependent variables, in considering the attrition of researchers from academia, roughly one in five respondents (or 21.6%, *n* = 955) had partners who intended to pursue a career in academia but ultimately did not, had applied for academic jobs but stopped, or had actually held an academic job but no longer did. We found that 33.7% (*n* = 322) of these partners left academia for personal reasons—either because they decided to prioritize their partner’s (*i.e.,* the respondent’s) academic career, family responsibilities, or both. The remaining 633 partners left due to professional reasons (*e.g.,* more appealing opportunities outside academia). Among those who left academia, 86.7% (*n* = 828) were employed when the data were collected (Table 1). Among those who were employed and reported an income (*n* = 744), the mean salary was 4.9 (*i.e.,* between “$75,000–$99,999” and “$100,000–$124,999” but close to the latter) for those who had left for professional reasons and 4.1 (*i.e.,* between “$75,000–$99,999” and “$100,000–$124,999” but close to the former) for those who had left for personal reasons (Table 2).

**Table 1.** Partners’ Characteristics

	Full sample		Attrition sample	
	No.	%	No.	%
Key independent variables				
*Partner’s gender*				
Women	1,983	44.8	447	46.8
Men	2,338	52.8	476	49.8
Other genders	104	2.4	32	3.4
*Partner’s race/ethnicity*				
White	3,082	69.7	636	66.6
Asian	629	14.2	146	15.3
Black	129	2.9	41	4.3
Hispanic	284	6.4	54	5.7
Not reported	192	4.3	51	5.3
Other races	109	2.5	27	2.8
*Partner’s field*				
Arts, humanities, social sciences	1,893	42.8	338	35.4
Natural sciences, engineering, or mathematics	1,289	29.1	218	22.8
Medicine, nursing, or health sciences	731	16.5	171	17.9
Professional fields	512	11.6	228	23.9
Dependent variables				
*Partner attrition reason and employment*				
Left academia due to personal reasons (*i.e.,* to prioritize their partner’s academic career, to prioritize family responsibilities, or both)	—	—	322	33.7
Left academia due to professional reasons (*e.g.,* more appealing jobs outside academia)	—	—	633	66.3
Partner currently employed (1 = yes)			828	86.7

**Table 2.** Mean Salary of Partners Who Have Left Academia and Reported an Income, by Attrition Reason

	No.	Mean	Standard deviation	Min	Max
Mean earnings for currently employed	744	4.7	1.9	1	7
Left academia due to professional reasons	518	4.9	1.9	1	7
Left academia due to personal reasons	226	4.1	2.0	1	7

#### Among all academic partners, who is leaving academia?

Regarding the key independent variables of partners’ gender, race, and field, we ran multinomial logistic regressions and found no differences based on gender or racial/ethnic group for partner’s attrition from academia (regardless of reason). However, partners who earned their highest degrees in medicine and professional fields were more likely to leave academia for professional reasons relative to remaining in academia, and partners with their highest degrees in STEM were more likely to leave academia for personal reasons relative to remaining in academia than those in the arts, humanities, and social sciences (Supplementary Table S4).

**FIG. 1.**
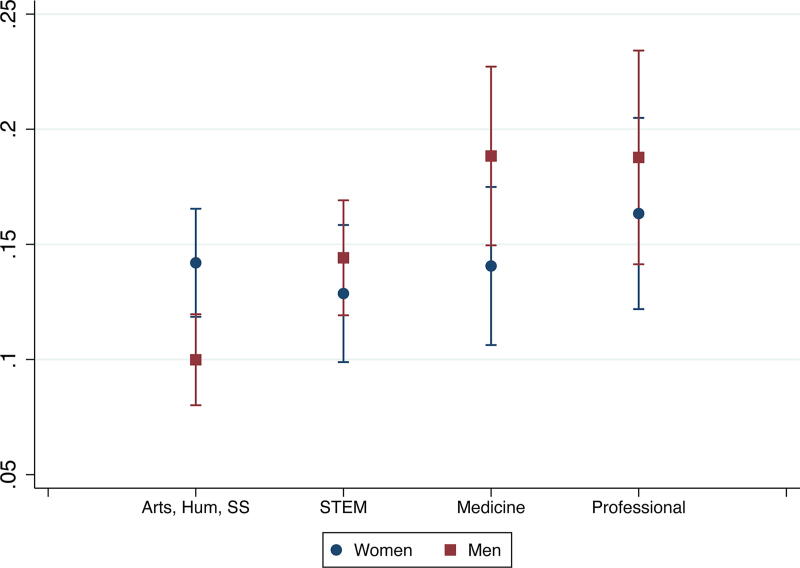
Probabilities of leaving academia due to professional reasons, by gender and field.

**FIG. 2.**
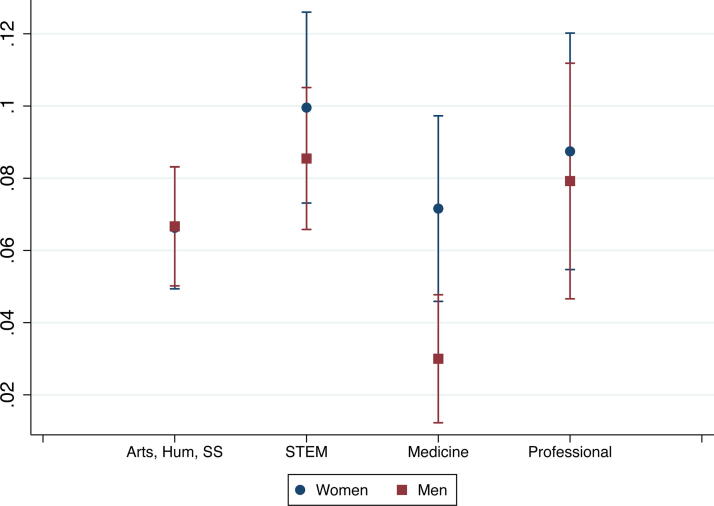
Probabilities of leaving academia due to personal reasons, by gender and field.

Further differences emerged when examining interaction effects. While we did not find significant interaction effects between gender and race, we found significant interaction effects between partners’ gender and field and between race and field (Supplementary Table S5). Since marginal effects better show how variables (*e.g.,* gender and field) interact in affecting outcomes (*e.g.,* leaving academia for personal reasons), we did analyses of marginal effects in addition to the interaction effects reported in Supplementary Table S5. Here, we focus on leaving academia for either reason instead of staying in academia. Figures 1 and 2 show gender gaps in some fields: women partners in medicine were more likely to leave academia for personal reasons than comparable men, and women partners in arts, humanities, and social sciences were more likely to leave academia for professional reasons than their men counterparts. We also found field differences among just men: compared with men partners in arts, humanities, and social sciences, men partners with their highest degree in STEM, medicine, and professional fields were more likely to leave academia for professional reasons, and men partners in medicine were also less likely to leave academia for personal reasons (Figs. 1 and 2). Men partners trained in arts, humanities, and social sciences had the lowest probability of leaving academia for professional reasons (10%), and men partners in medicine had the lowest probability of leaving academia for personal reasons (3%) among all gender and field groups. Among women, the field differences in attrition patterns were not statistically significant.

**FIG. 3.**
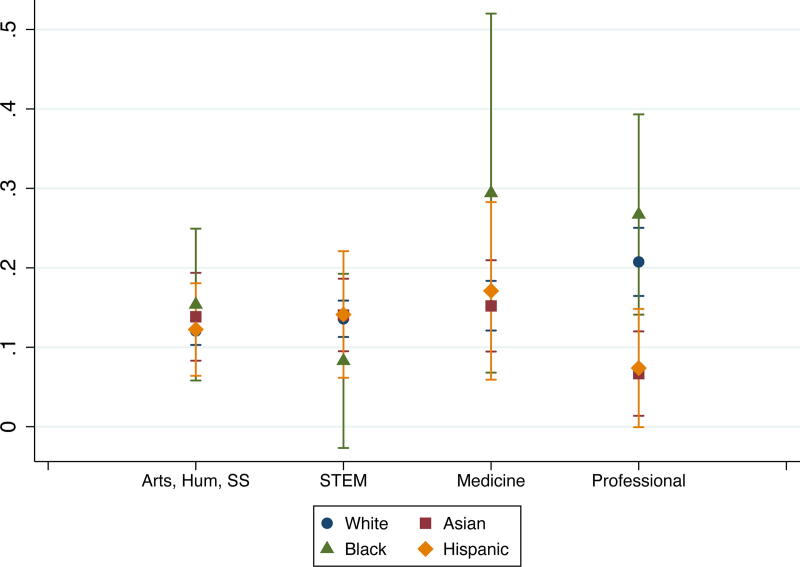
Probabilities of leaving academia due to professional reasons, by race and field.

**FIG. 4.**
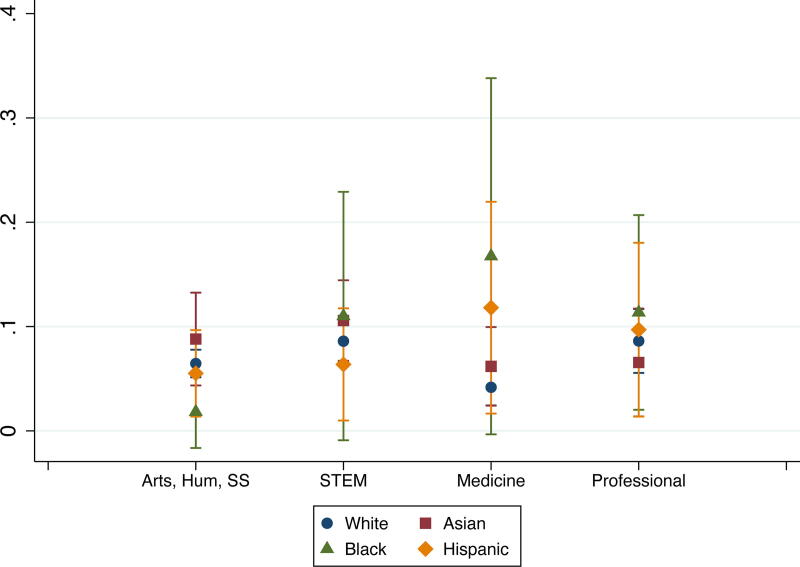
Probabilities of leaving academia due to personal reasons, by race and field.

In terms of the marginal effects of partners’ race and field, we found field differences among Whites. Compared with their counterparts in arts, humanities, and social sciences, Whites in medicine were less likely to leave academia for personal reasons, and Whites in professional fields were more likely to leave for professional reasons. We also found racial differences among some fields. Compared with their White counterparts, Asians and Hispanics in professional fields were less likely to leave for professional reasons, and Blacks in arts, humanities, and social sciences were less likely to leave for personal reasons (Figs. 3 and 4). In fact, Asian and Hispanic partners had lower probabilities of leaving academia for professional reasons (6.8% and 7.5%, respectively) than Black and White partners (27% and 21%, respectively). These findings highlight multiple racial and field interactions. While Blacks in medicine had low probability of staying in academia and high probabilities of leaving academia due to professional or personal reasons, the findings compared with those of other groups were not statistically significant due to the wide confidence intervals based on small sample sizes.

#### What effect does leaving academia have on partners’ career outcomes?

Partners left academia for different reasons, and regression results show that attrition reasons impact career outcomes. Among the partners who left academia, those who left due to personal reasons were less likely to be currently employed (Supplementary Table S6, Model 1) and, if employed, were paid less than those who left for professional reasons (Supplementary Table S7, Model 1). Also, men who left academia were more likely to be employed and paid more than their women counterparts. We did not find racial/ethnic differences in either outcome, but compared with arts, humanities, and social sciences, those with their highest degree in STEM, medicine, and professional fields were more likely to be currently employed and paid more (Supplementary Tables S6 and S7, Model 1).

**FIG. 5.**
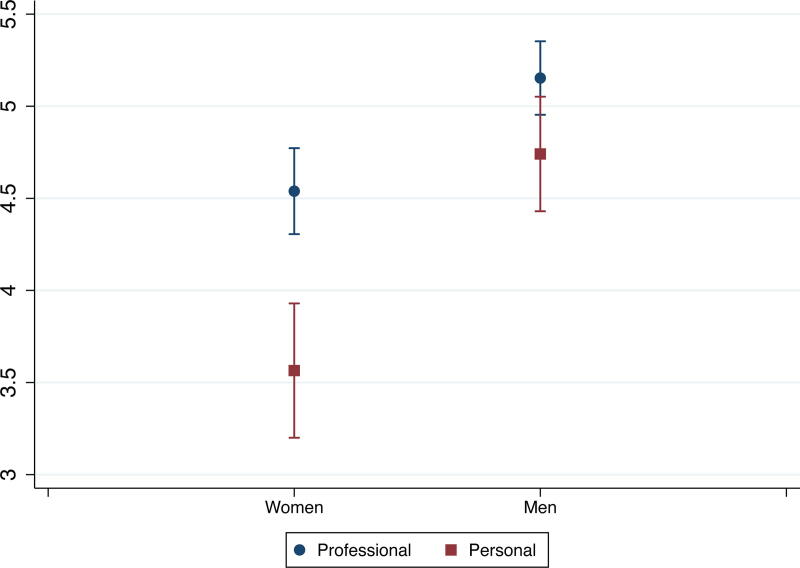
Predictive margins of partner’s gender and attrition reason.

**FIG. 6.**
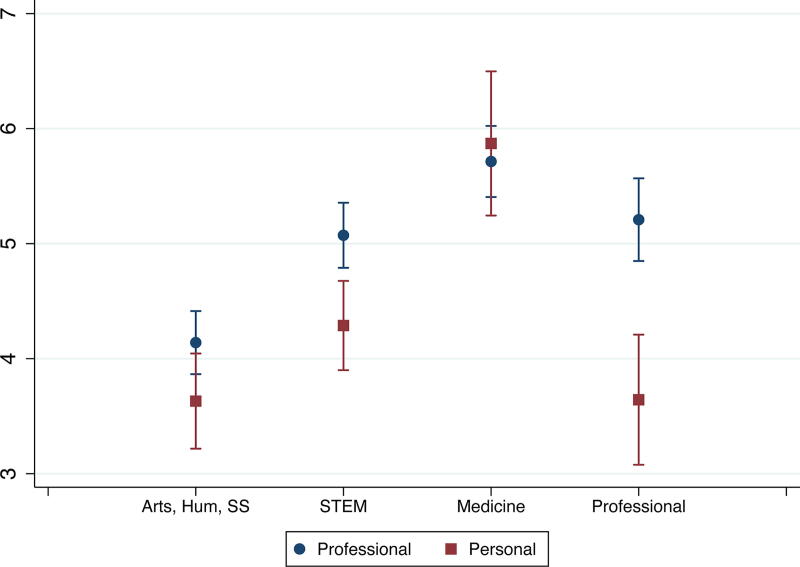
Predictive margins of partner’s field and attrition reason.

Additionally, we did not find any interaction effects for partners’ employment status (Supplementary Table S6, Models 2–4), but in terms of current pay, the interaction effect of attrition reason and gender and that of attrition reason and field were significant (Supplementary Table S7, Models 2–4). Further analyses of marginal effects revealed that men made more than women, irrespective of attrition reasons, but the gender pay gap was greater among those who left due to personal reasons. Also, the salary penalty associated with attrition due to personal reasons was found among both men and women but greater among women than men. In other words, women who left academia due to personal reasons were among the lowest paid—less than women leaving academia for professional reasons and men leaving academia for both reasons (Fig. 5). Furthermore, we found that compared with their counterparts in arts, humanities, and social sciences, partners trained in STEM and medicine were paid more, despite attrition reasons, and those in professional fields were paid more if they had left academia due to professional reasons. Also, compared with those who left academia for professional reasons, those who left for personal reasons were paid less in three fields: arts, humanities, and social sciences; STEM; and professional fields (Fig. 6). In other words, leaving academia for personal reasons disadvantaged partners in all fields, relative to leaving for professional reasons, except for medicine, and the pay gap due to attrition reasons was the greatest in professional fields.

## Discussion

In this study, we set out to understand who might leave academia because of their relationship status as part of an academic couple and what the consequences of that decision are for their careers. We found that 22% of our survey respondents had a partner who was an academic or aspiring to be one but ended up leaving that career path. This is a substantial number given that 36% of academics, more generally, have an academic partner.^7^

While there are many reasons someone may decide to leave academia, our survey found that one-third of the partners who decided to leave academia left because of personal reasons, specifically to prioritize their partner’s (*i.e.,* the respondent’s) academic career, family responsibilities, or both. When a partner stops pursuing academic employment to prioritize the respondent’s academic career, we likely see the direct effect of the dual-career relationship on such decisions. As previous literature has shown, academic couples face numerous obstacles to pursuing their careers, with many feeling that they must compromise one person’s career to support the other or their family.^13,16,31–34^ If academic institutions did more for dual-career academic couples by providing them with positions that appropriately supported both members’ careers, it is possible that there would be less attrition because partners would not have to give up or alter their own professional goals.

Our analysis also revealed that field had important effects on attrition. Specifically, partners in medicine and professional fields were more likely to leave academia for professional reasons, and those in STEM were more likely to leave academia for personal reasons relative to staying in academia. Given that medicine and professional fields primarily train students for non-academic careers, it may be no surprise that many did not ultimately end up in academic positions. However, our investigation focused on those partners who wanted, at least at some point, an academic position. Likewise, partners who were trained in STEM fields were also more likely to leave academia than to stay in academia, compared with partners in the arts, humanities, and social sciences. These findings, again, likely reflect more professional opportunities outside of academia for those in STEM than those in the arts, humanities, and social sciences. Nonetheless, these results mean that people who were partnered with an academic and trained in STEM fields were leaving academia despite their intent to pursue academic careers, and notably, many were doing so to prioritize the academic career of their partner (*i.e.,* the respondent) and/or to prioritize family responsibilities. Thus, the leaky pipeline in STEM fields appears to be influenced by decisions being made by dual-career academics to abandon one academic career.

We did not find gender differences in, or interaction effects of gender and race on, partners’ attrition. Given findings in the extant literature about the importance of gender, especially in regard to career sacrifice, it was unexpected that gender did not play a more central role in attrition.^11–13,15^ However, we found interaction effects between gender and field. Compared to their men counterparts, women in medicine were more likely to leave academia for personal reasons than comparable men, and women in arts, humanities, and social sciences were more likely to leave academia for professional reasons. Among men, those with their highest degree in STEM, medicine, or professional fields were more likely to leave academia for professional reasons than those with their highest degrees in arts, humanities, and social sciences. Men in medicine had the lowest probability of leaving academia for personal reasons among all gender and field groups. These findings indicate that women in medicine may be sacrificing a desired academic career to prioritize their partner’s career or family responsibilities. By contrast, all the other gender/field combinations showed decisions to leave academia for professional reasons, such as better job opportunities outside of academia or changed attitudes about an academic career, for which lack of success at securing two positions or disillusionment about academia could be factors.

Also, while there were no overall differences in attrition by race, we found interaction effects between race and field. In professional fields, Black and White partners (27% and 21%, respectively) had very high probabilities of leaving academia for professional reasons, while Asians (6.8%) and Hispanics (7.5%) had lower probabilities of leaving academia for professional reasons. Although these findings may not be the result of these individuals being part of an academic couple, future research could explore the intersection of how professional reasons for leaving academia may differ between researchers from underrepresented minoritized groups who have academic partners and those who do not. Given the ongoing concerns about the attrition of researchers of color from academic careers,^35–38^ this finding suggests that universities’ partner-hiring mechanisms could be a critical avenue to assist with their diversity efforts with the added benefit of supporting the academic ambitions of partners of color.

Our study also emphasizes that the reasons why someone leaves academia matter for their longer-term career and financial situation. Our results showed that partners who left academia for personal reasons were less likely to be employed in any job and, among those who were employed and reported earnings, were paid less than partners whose attrition was for professional reasons. Not surprisingly, those in STEM, medicine, and professional fields had higher rates of non-academic employment and higher salaries compared to partners whose degrees were in the arts, humanities, and social sciences. Our results also reveal a substantial overall gender effect with men who left academia being more likely to be employed and having higher wages than women. Additionally, women who left academia due to personal reasons were making the least among the four gender and attrition groups (*i.e.,* making less than women leaving for professional reasons and men leaving academia for either reason); their earnings were penalized not only due to their gender but because of the reason for their attrition. While this result may be expected given the persistence of the gender wage gap and gendered patterns of caregiving,^12,39–42^ it nonetheless highlights the continued severe consequences to women of changing careers or leaving the workforce for personal reasons, even among women who hold advanced degrees.

Our findings also demonstrate how field and attrition reasons intersect to affect earnings among those who have left academia. While leaving academia for personal reasons hurt partners’ earnings across three of the four fields, such a disadvantage was especially great among partners trained in professional fields leaving academia for personal reasons—they were among the lowest paid, and the earnings gap due to attrition reason was the greatest in professional fields. These findings continue to point to the disadvantages of professionals who sacrifice their careers for family, and such a disadvantage is heightened in a field where earnings outside academia are expected to be high. Future research can continue to track the challenges of academic partners, particularly in professional fields. While partners who left academic medicine were not at a disadvantage in terms of employment or wages, the fact that women were more likely than men to leave academic medicine for personal reasons further indicates that gender is playing an important role in attrition. These findings also suggest that if universities and colleges accommodate partners’ careers, it is not only beneficial to the institutions in terms of not losing talent but also to the employees who can then pursue a career that may also be more compatible with fulfilling family responsibilities.^43,44^

There are limitations to our study. First, the primary focus of the survey was on the respondent and their career trajectory. As a result, we did not ask questions about the timing of their partner’s decision to stop looking for an academic job. They may have recently stopped looking or made the decision to find employment outside of academia decades prior. Moreover, it is the respondents, and not the partners themselves, who have reported on why the partners left academia, which assumes that they understood and accurately reported their partner’s reasons. It is also difficult to tease out the weight that personal versus professional factors may have had on partners’ decisions to leave academia, especially in cases where both types of reason were at play. For analytic purposes, we treated these reasons as distinct, but in practice, personal and professional reasons are likely intertwined. Another limitation is the small sample size of some groups, including genders other than men and women and some racial/ethnic groups, such as Black partners. These groups are small as a whole and even smaller when data are disaggregated by field, and as a result, some findings pertaining to these groups (*e.g.,* Black partners in medicine) are not statistically significant. Additionally, the overwhelming majority of couples (92%) were in heteronormative relationships, making it difficult to capture any differences in attrition for LGBTQ+ scholars.

## Conclusions

Together, our analyses of these patterns of attrition from the academy are indicative of several notable trends. First, an important percentage of people who intend to pursue academic careers ultimately do not do so, with many changing their career to support their academic partner or to prioritize family responsibilities. Second, some fields, including STEM, medicine, and other professional fields, are more prone to attrition than others in the context of dual-career academic decision making. Third, and perhaps most important, there are gendered and racialized patterns of attrition that contribute to the academy being less diverse. This is particularly striking for women in medicine who leave for personal reasons. These trends indicate that the choices made by dual-career (or aspiring dual-career) couples in response to the academic job market and to institutions’ policies for partner hiring have substantial effects on the demographic makeup of academia. It also suggests that universities are losing talented scholars who would otherwise contribute to the academic workforce. At the same time, we found that there are profoundly negative career and salary consequences to individuals, especially women, who leave academia for personal reasons. By supporting both the personal and professional needs of academic couples, universities have the opportunity to make their own institutions more diverse and to patch a major hole in the leaky pipeline.

## Abbreviations Used

LGBTQ+: lesbian, gay, bisexual, transgender, queer or questioning, and more
RQ1: research question 1
RQ2: research question 2
STEM: science, technology, engineering, and mathematics
