# Supplementary material for: Leaving Academia: Dual-Career Relationships and Partners’ Attrition from Academic Careers
Source: Womens Health Rep (New Rochelle). 2025 Aug 11;6(1):752–62. doi: 10.1177/26884844251366373 (PMC12415177; doi:10.1177/26884844251366373)
Supplement: Supplementary Tables [file 26884844251366373_supplementary_tables.docx]

**Supplementary Materials for**

Leaving Academia: Dual-Career Relationships and Partners’ Attrition from Academic Careers

**Table S1. 100 Included Institutions by Carnegie Classification, Region, and Control**

| **Institution** | **Carnegie Classification** | **Region** | **Control** |
| --- | --- | --- | --- |
| Washington University in St Louis | R1 | Midwest | Private |
| Ohio State University | R1 | Midwest | Public |
| University of Wisconsin-Madison | R1 | Midwest | Public |
| Carnegie Mellon University | R1 | Northeast | Private |
| Johns Hopkins University | R1 | Northeast | Private |
| Tufts University | R1 | Northeast | Private |
| University of Massachusetts-Amherst | R1 | Northeast | Public |
| University of New Hampshire | R1 | Northeast | Public |
| Emory University | R1 | Southeast | Private |
| University of Florida | R1 | Southeast | Public |
| University of Louisville | R1 | Southeast | Public |
| University of South Carolina | R1 | Southeast | Public |
| University of South Florida | R1 | Southeast | Public |
| University of Southern California | R1 | West | Private |
| Colorado School of Mines | R1 | West | Public |
| University of California-Irvine | R1 | West | Public |
| University of Nevada-Reno | R1 | West | Public |
| University of North Texas | R1 | West | Public |
| Illinois Institute of Technology | R2 | Midwest | Private |
| Oakland University | R2 | Midwest | Public |
| Southern Illinois University-Carbondale | R2 | Midwest | Public |
| University of Missouri-St Louis | R2 | Midwest | Public |
| Seton Hall University | R2 | Northeast | Private |
| Teachers College at Columbia University | R2 | Northeast | Private |
| Morgan State University | R2 | Northeast | Public |
| University of Massachusetts-Dartmouth | R2 | Northeast | Public |
| Nova Southeastern University | R2 | Southeast | Private |
| North Carolina A & T State University | R2 | Southeast | Public |
| Tennessee State University | R2 | Southeast | Public |
| University of Arkansas at Little Rock | R2 | Southeast | Public |
| Claremont Graduate University | R2 | West | Private |
| California State University-Fresno | R2 | West | Public |
| Sam Houston State University | R2 | West | Public |
| The University of Texas Rio Grande Valley | R2 | West | Public |
| Graceland University-Lamoni | Larger-masters | Midwest | Private |
| John Carroll University | Larger-masters | Midwest | Private |
| Lakeland University | Larger-masters | Midwest | Private |
| Lawrence Technological University | Larger-masters | Midwest | Private |
| North Park University | Larger-masters | Midwest | Private |
| Upper Iowa University | Larger-masters | Midwest | Private |
| Purdue University Northwest | Larger-masters | Midwest | Public |
| University of Central Missouri | Larger-masters | Midwest | Public |
| University of Wisconsin-Stout | Larger-masters | Midwest | Public |
| Alfred University | Larger-masters | Northeast | Private |
| Bryant University | Larger-masters | Northeast | Private |
| Champlain College | Larger-masters | Northeast | Private |
| Fairleigh Dickinson University-Florham Campus | Larger-masters | Northeast | Private |
| Fairleigh Dickinson University- Metropolitan Campus | Larger-masters | Northeast | Private |
| Johnson & Wales University | Larger-masters | Northeast | Private |
| McDaniel College | Larger-masters | Northeast | Private |
| The College of Saint Rose | Larger-masters | Northeast | Private |
| Central Connecticut State University | Larger-masters | Northeast | Public |
| Fitchburg State University | Larger-masters | Northeast | Public |
| Kutztown University of Pennsylvania | Larger-masters | Northeast | Public |
| Southern Connecticut State University | Larger-masters | Northeast | Public |
| SUNY Polytechnic Institute | Larger-masters | Northeast | Public |
| Lenoir-Rhyne University | Larger-masters | Southeast | Private |
| Stetson University | Larger-masters | Southeast | Private |
| The University of Tampa | Larger-masters | Southeast | Private |
| Coastal Carolina University | Larger-masters | Southeast | Public |
| Georgia College & State University | Larger-masters | Southeast | Public |
| Northwestern State University of Louisiana | Larger-masters | Southeast | Public |
| Golden Gate University | Larger-masters | West | Private |
| Middlebury Institute of International Studies at Monterey | Larger-masters | West | Private |
| University of Dallas | Larger-masters | West | Private |
| Wayland Baptist University | Larger-masters | West | Private |
| California State University-Los Angeles | Larger-masters | West | Public |
| California State University-Sacramento | Larger-masters | West | Public |
| East Central University | Larger-masters | West | Public |
| The University of Texas Permian Basin | Larger-masters | West | Public |
| University of Washington-Bothell Campus | Larger-masters | West | Public |
| Western New Mexico University | Larger-masters | West | Public |
| College of Saint Benedict | Small liberal arts | Midwest | Private |
| Denison University | Small liberal arts | Midwest | Private |
| Divine Word College | Small liberal arts | Midwest | Private |
| Grinnell College | Small liberal arts | Midwest | Private |
| Kalamazoo College | Small liberal arts | Midwest | Private |
| Kenyon College | Small liberal arts | Midwest | Private |
| Macalester College | Small liberal arts | Midwest | Private |
| Colgate University | Small liberal arts | Northeast | Private |
| Emmanuel College | Small liberal arts | Northeast | Private |
| Houghton College | Small liberal arts | Northeast | Private |
| Muhlenberg College | Small liberal arts | Northeast | Private |
| Saint Anselm College | Small liberal arts | Northeast | Private |
| Trinity College | Small liberal arts | Northeast | Private |
| Wells College | Small liberal arts | Northeast | Private |
| Williams College | Small liberal arts | Northeast | Private |
| SUNY at Purchase College | Small liberal arts | Northeast | Public |
| Centre College | Small liberal arts | Southeast | Private |
| Covenant College | Small liberal arts | Southeast | Private |
| Guilford College | Small liberal arts | Southeast | Private |
| Hendrix College | Small liberal arts | Southeast | Private |
| Meredith College | Small liberal arts | Southeast | Private |
| Warren Wilson College | Small liberal arts | Southeast | Private |
| Washington and Lee University | Small liberal arts | Southeast | Private |
| New College of Florida | Small liberal arts | Southeast | Public |
| St. John's College | Small liberal arts | West | Private |
| Thomas Aquinas College | Small liberal arts | West | Private |
| University of Puget Sound | Small liberal arts | West | Private |
| Whittier College | Small liberal arts | West | Private |

**Table S2. Numbers and Frequencies of Control Variables**

|  | **Full Sample** | | **Attrition Sample** | |
| --- | --- | --- | --- | --- |
|  | No. | % | No. | % |
| Respondent and partner in same field | 2,003 | 45.3 | 301 | 31.5 |
| *Partner’s Birthplace* |  |  |  |  |
| In the U.S. | 2,949 | 66.6 | 642 | 67.2 |
| Outside the U.S. | 1,349 | 30.5 | 285 | 29.8 |
| Not reported or unknown | 127 | 2.9 | 28 | 2.9 |
| *Partner’s Years Since Highest Degree* | | | | |
| Up to 5 years | 383 | 8.7 | 120 | 12.6 |
| 6-10 | 733 | 16.6 | 171 | 17.9 |
| 11-15 | 728 | 16.5 | 162 | 17.0 |
| 16-20 | 627 | 14.2 | 132 | 13.8 |
| 21-25 | 476 | 10.8 | 91 | 9.5 |
| 26-30 | 408 | 9.2 | 84 | 8.8 |
| 31-35 | 293 | 6.6 | 63 | 6.6 |
| 36-40 | 235 | 5.3 | 40 | 4.2 |
| 41 or more years | 289 | 6.5 | 35 | 3.7 |
| Year not reported or unknown | 253 | 5.7 | 57 | 6.0 |
| *Respondent’s Perceived Biggest Obstacle to Hiring Academic Couples* | | | | |
| Lack of funding | 1,191 | 26.9 | 287 | 30.1 |
| Lack of support from university leadership | 949 | 21.5 | 158 | 16.5 |
| Lack of good fit or match | 857 | 19.4 | 204 | 21.4 |
| Lack of a formal partner-hire policy | 654 | 14.8 | 153 | 16.0 |
| Other obstacles | 774 | 17.5 | 153 | 16.0 |
| *Carnegie Classification of Respondent’s Institution* | | | | |
| R1 | 2,963 | 67.0 | 620 | 64.9 |
| R2 | 413 | 9.3 | 94 | 9.8 |
| Master’s | 512 | 11.6 | 133 | 13.9 |
| Liberal Arts | 537 | 12.1 | 108 | 11.3 |
| *Respondent’s Years at Current Institution* | | | | |
| Less than 1 year | 231 | 5.2 | 57 | 6.0 |
| 1-5 years | 1,152 | 26.1 | 266 | 27.9 |
| 5-10 years | 991 | 22.4 | 208 | 21.8 |
| 10-20 years | 1,194 | 27.0 | 256 | 26.8 |
| More than 20 years | 855 | 19.3 | 168 | 17.6 |
| *Respondent’s Region* | | | | |
| Midwest | 1,344 | 30.4 | 250 | 26.2 |
| Northeast | 995 | 22.5 | 235 | 24.6 |
| Southeast | 965 | 21.8 | 215 | 22.5 |
| West | 1,121 | 25.3 | 255 | 26.7 |
| *Respondent’s Location* | | | | |
| City | 3,099 | 70.0 | 666 | 69.7 |
| Rural | 22 | 0.5 | 3 | 0.3 |
| Suburb | 906 | 20.5 | 212 | 22.2 |
| Town | 398 | 9.0 | 74 | 7.8 |
| *Partner’s Sexual Orientation* | | | | |
| Heterosexual | 3,885 | 87.8 | 817 | 85.5 |
| Bisexual | 130 | 2.9 | 41 | 4.3 |
| Gay, lesbian, queer | 202 | 4.6 | 41 | 4.3 |
| Self-describe/not to report | 208 | 4.7 | 56 | 5.9 |
| *Couple Type* | | | | |
| Heteronormative | 4,095 | 92.5 | 880 | 92.1 |
| Non-heteronormative | 247 | 5.6 | 50 | 5.2 |
| Couple type not reported or unknown | 83 | 1.9 | 25 | 2.6 |

**Table S3. Mean Values of Control Variables (Continuous Variables, with Values 0-1)**

|  | **Full Sample** | | | **Attrition Sample** | | |
| --- | --- | --- | --- | --- | --- | --- |
|  | **No.** | **Mean** | **Std. dev.** | **No.** | **Mean** | **Std. dev.** |
| No. of children | 4,425 | 0.37 | 0.29 | 955 | 0.40 | 0.30 |
| Respondent’s agreement with: Academic couples who get two positions at the same university are more likely to remain at that institution. | 4,422 | 0.91 | 0.16 | 955 | 0.88 | 0.18 |
| Respondent’s agreement with: Departments regularly exclude faculty job candidates who are known to have academic partners. | 4,416 | 0.41 | 0.26 | 952 | 0.38 | 0.25 |
| Respondent’s agreement with: Partner hiring often results in academic positions being created for less competitive partner candidates. | 4,418 | 0.52 | 0.27 | 952 | 0.58 | 0.25 |
| Respondent’s agreement with: Partner hiring allows departments to gain additional faculty positions that they would not have otherwise. | 4,419 | 0.66 | 0.25 | 952 | 0.62 | 0.25 |
| Respondent’s agreement with: In general, there is a stigma attached to being a partner hire if the couple is in the same department. | 4,418 | 0.70 | 0.25 | 952 | 0.71 | 0.23 |

**Table S4. Multinomial Logistic Regression Results Showing Effects of Partner’s Gender, Race, and Field on Attrition**

|  |  | **Professional** | **Personal** | |
| --- | --- | --- | --- | --- |
| *Partner’s Gender (Reference: Women)* | | | |  |
| Men |  | -0.0307 | -0.199 | |
|  |  | (0.0934) | (0.123) | |
| Other Genders |  | 0.361 | -0.465 | |
|  |  | (0.302) | (0.560) | |
| *Partner’s Race/Ethnicity (Reference: White)* | | | |  |
| Asian |  | -0.0786 | 0.189 | |
|  |  | (0.151) | (0.186) | |
| Black |  | 0.380 | 0.256 | |
|  |  | (0.233) | (0.321) | |
| Hispanic |  | -0.108 | 0.0330 | |
|  |  | (0.201) | (0.253) | |
| Not Reported |  | 0.0159 | 0.403 | |
|  |  | (0.249) | (0.311) | |
| Other Races |  | 0.336 | 0.00390 | |
|  |  | (0.263) | (0.417) | |
| *Partner’s Field (Reference: Arts, Humanities, and Social Sciences)* | | | |  |
| STEM |  | 0.206 | 0.406** | |
|  |  | (0.115) | (0.145) | |
| Medicine |  | 0.343** | -0.222 | |
|  |  | (0.129) | (0.199) | |
| Professional |  | 0.483*** | 0.354 | |
|  |  | (0.140) | (0.188) | |
| *Partner’s Birthplace (Reference: U.S.-born)* | | | |  |
| Not U.S.-born |  | -0.173 | -0.200 | |
|  |  | (0.114) | (0.150) | |
| Not reported |  | -0.215 | -0.997 | |
|  |  | (0.287) | (0.510) | |
| *Partner’s Years Since Highest Degree (Reference: Up to 5 years)* | | | |  |
| 6-10 years |  | -0.452** | -0.260 | |
|  |  | (0.175) | (0.218) | |
| 11-15 |  | -0.599*** | -0.235 | |
|  |  | (0.180) | (0.220) | |
| 16-20 |  | -0.608*** | -0.541* | |
|  |  | (0.183) | (0.235) | |
| 21-25 |  | -0.705*** | -0.731** | |
|  |  | (0.199) | (0.264) | |
| 26-30 |  | -0.552** | -0.776** | |
|  |  | (0.204) | (0.281) | |
| 31-35 |  | -0.427* | -0.888** | |
|  |  | (0.214) | (0.323) | |
| 36-40 |  | -0.787** | -1.535*** | |
|  |  | (0.246) | (0.427) | |
| 41 or more years |  | -1.229*** | -2.392*** | |
|  |  | (0.257) | (0.544) | |
| Year unknown |  | -0.654** | -0.429 | |
|  |  | (0.246) | (0.306) | |
| *Partner in same field* |  | -0.692*** | -0.643*** | |
|  |  | (0.0966) | (0.128) | |
| *Agreement w/remaining* |  | -1.557*** | -0.453 | |
|  |  | (0.252) | (0.395) | |
| *Agreement w/excluding* |  | -0.292 | -0.477 | |
|  |  | (0.190) | (0.256) | |
| *Agreement w/less competitive* |  | 0.681*** | 1.088*** | |
|  |  | (0.177) | (0.245) | |
| *Agreement w/add. positions* |  | -0.705*** | -0.428 | |
|  |  | (0.178) | (0.250) | |
| *Agreement w/stigma* |  | 0.197 | 0.284 | |
|  |  | (0.187) | (0.249) | |
| *Perceived Biggest Obstacles to Academic Couple Hiring (Reference: Lack of Funding)* | | | |  |
| Lack of leadership support |  | -0.292* | -0.311 | |
|  |  | (0.139) | (0.176) | |
| Lack of good fit or match |  | -0.142 | -0.268 | |
|  |  | (0.130) | (0.182) | |
| Lack of partner-hire policy |  | 0.0547 | -0.00682 | |
|  |  | (0.145) | (0.183) | |
| Other obstacles |  | -0.136 | -0.395* | |
|  |  | (0.138) | (0.193) | |
| *Partner’s Sexual Orientation (Reference: Heterosexual)* | | | |  |
| Bisexual |  | 0.583* | 0.0795 | |
|  |  | (0.242) | (0.352) | |
| Gay, lesbian, queer |  | -0.142 | -0.346 | |
|  |  | (0.218) | (0.311) | |
| Others |  | 0.273 | 0.0418 | |
|  |  | (0.232) | (0.340) | |
| Constant |  | 0.537 | -1.316** | |
|  |  | (0.348) | (0.485) | |
| N |  | 4,399 | 4,399 | |

Robust standard errors in parentheses

*** p<0.001, ** p<0.01, * p<0.05

**Table S5. Interaction Effects of Partner’s Gender, Race, and Field on Attrition**

|  | **Professional** | **Personal** |
| --- | --- | --- |
| ***1^st^ Regression: Partner Gender and Race Interaction*** | | |
| Women * Asian | 0.0832 | 0.243 |
|  | (0.191) | (0.234) |
| Women * Black | 0.169 | 0.126 |
|  | (0.341) | (0.461) |
| Women * Hispanic | -0.0581 | 0.394 |
|  | (0.297) | (0.325) |
| Men * White | 0.0299 | -0.167 |
|  | (0.112) | (0.150) |
| Men * Asian | -0.225 | -0.0465 |
|  | (0.227) | (0.268) |
| Men * Black | 0.488 | 0.201 |
|  | (0.330) | (0.448) |
| Men * Hispanic | -0.0429 | -0.823 |
|  | (0.273) | (0.454) |
| ***2^nd^ Regression: Partner Gender and Field Interaction*** | | |
| Women * STEM | -0.0677 | 0.444* |
|  | (0.177) | (0.214) |
| Women * Medicine | -0.00360 | 0.0844 |
|  | (0.186) | (0.253) |
| Women * Professional | 0.212 | 0.350 |
|  | (0.195) | (0.262) |
| Men * Arts, Hum, SS | -0.422** | -0.0596 |
|  | (0.155) | (0.199) |
| Men * STEM | 0.0499 | 0.292 |
|  | (0.152) | (0.199) |
| Men * Medicine | 0.303 | -0.781* |
|  | (0.174) | (0.349) |
| Men * Professional | 0.380 | 0.277 |
|  | (0.196) | (0.278) |
| ***3^rd^ Regression: Partner Race and Field Interaction*** | | |
| White * STEM | 0.179 | 0.348* |
|  | (0.137) | (0.175) |
| White * Medicine | 0.250 | -0.424 |
|  | (0.157) | (0.253) |
| White * Professional | 0.729*** | 0.471* |
|  | (0.167) | (0.236) |
| Asian * Arts, Hum, SS | 0.205 | 0.379 |
|  | (0.267) | (0.319) |
| Asian * STEM | 0.254 | 0.599* |
|  | (0.228) | (0.255) |
| Asian * Medicine | 0.280 | 0.00266 |
|  | (0.260) | (0.364) |
| Asian * Professional | -0.677 | -0.0658 |
|  | (0.460) | (0.464) |
| Black * Arts, Hum, SS | 0.225 | -1.291 |
|  | (0.405) | (1.002) |
| Black * STEM | -0.367 | 0.553 |
|  | (0.768) | (0.660) |
| Black * Medicine | 1.411* | 1.476* |
|  | (0.643) | (0.721) |
| Black * Professional | 1.146** | 0.912 |
|  | (0.368) | (0.516) |
| Hispanic * Arts, Hum, SS | 0.00251 | -0.171 |
|  | (0.302) | (0.432) |
| Hispanic * STEM | 0.191 | 0.0178 |
|  | (0.367) | (0.488) |
| Hispanic * Medicine | 0.525 | 0.783 |
|  | (0.449) | (0.551) |
| Hispanic * Professional | -0.518 | 0.389 |
|  | (0.579) | (0.518) |

Notes: These results were pooled from three separate regressions. The independent and control variables were included in the regressions, but the main effects are not shown in this table. This table shows only the coefficients with robust standard errors in parentheses.

**Table S6. OLS Regression Results Showing Effects of Partner’s Attrition Reasons and Interaction Effects on Employment Status**

|  | (1) | (2) | (3) | (4) |
| --- | --- | --- | --- | --- |
| Personal reasons | -1.372*** | -1.365*** | -1.525*** | -1.220*** |
|  | (0.218) | (0.276) | (0.291) | (0.322) |
| *Partner Gender (Reference: Women)* |  |  |  |  |
| Men | 0.812*** | 0.816* | 0.783*** | 0.794*** |
|  | (0.215) | (0.337) | (0.213) | (0.216) |
| Other genders | 2.094* | 2.262 | 1.502 | 2.025* |
|  | (0.844) | (1.411) | (1.001) | (0.843) |
| *Partner Race/Ethnicity (Reference: White)* |  |  |  |  |
| Asian | -0.492 | -0.491 | -0.813* | -0.496 |
|  | (0.279) | (0.279) | (0.389) | (0.281) |
| Black | -0.369 | -0.370 | -0.0413 | -0.353 |
|  | (0.493) | (0.492) | (0.826) | (0.511) |
| Hispanic | -0.133 | -0.133 | -0.357 | -0.114 |
|  | (0.408) | (0.408) | (0.573) | (0.403) |
| Not reported | -0.0287 | -0.0269 | 0.812 | -0.0487 |
|  | (0.618) | (0.617) | (1.238) | (0.622) |
| Other races | -0.0879 | -0.0779 | -0.791 | -0.110 |
|  | (0.676) | (0.702) | (0.705) | (0.674) |
| *Partner Field (Reference: Arts, Humanities, and Social Sciences)* | | | | |
| STEM | 0.842** | 0.842** | 0.880** | 0.917* |
|  | (0.271) | (0.272) | (0.269) | (0.403) |
| Medicine | 0.682* | 0.682* | 0.712* | 0.905* |
|  | (0.316) | (0.316) | (0.324) | (0.453) |
| Professional | 0.788* | 0.789* | 0.795* | 0.908 |
|  | (0.355) | (0.352) | (0.353) | (0.529) |
| *Partner Nativity (Reference: U.S.-born)* |  |  |  |  |
| Not U.S.-born | -0.455* | -0.456* | -0.451 | -0.442 |
|  | (0.230) | (0.229) | (0.231) | (0.233) |
| Nativity not reported | -0.0232 | -0.0454 | 0.0338 | -0.0442 |
|  | (0.959) | (0.903) | (0.969) | (0.965) |
| *Partner Years Since Degree (Reference: Up to 5 years)* | | | | |
| 6-10 years | 0.630 | 0.627 | 0.612 | 0.635 |
|  | (0.361) | (0.365) | (0.359) | (0.359) |
| 11-15 | 0.706 | 0.707 | 0.653 | 0.709 |
|  | (0.377) | (0.380) | (0.381) | (0.378) |
| 16-20 | 0.181 | 0.180 | 0.150 | 0.193 |
|  | (0.395) | (0.395) | (0.394) | (0.391) |
| 21-25 | 1.117* | 1.114* | 1.109* | 1.125* |
|  | (0.525) | (0.524) | (0.530) | (0.532) |
| 26-30 | 0.447 | 0.445 | 0.410 | 0.473 |
|  | (0.538) | (0.538) | (0.547) | (0.538) |
| 31-35 | 0.280 | 0.277 | 0.234 | 0.304 |
|  | (0.582) | (0.585) | (0.600) | (0.586) |
| 36-40 | 0.380 | 0.376 | 0.281 | 0.390 |
|  | (0.616) | (0.618) | (0.618) | (0.618) |
| 41 or more years | 0.900 | 0.900 | 0.846 | 0.890 |
|  | (0.694) | (0.697) | (0.726) | (0.698) |
| Year unknown | 0.862 | 0.856 | 0.764 | 0.919 |
|  | (0.619) | (0.630) | (0.606) | (0.620) |
| *Partner in same field* | 0.325 | 0.325 | 0.298 | 0.336 |
|  | (0.237) | (0.237) | (0.238) | (0.241) |
| *No. of children* | -0.319 | -0.318 | -0.303 | -0.320 |
|  | (0.359) | (0.359) | (0.366) | (0.364) |
| *Carnegie Classification of Respondent’s Institution* (*Reference: Research I)* | | | | |
| R2 | -0.141 | -0.137 | -0.141 | -0.138 |
|  | (0.339) | (0.343) | (0.345) | (0.344) |
| Master’s | 0.267 | 0.271 | 0.314 | 0.246 |
|  | (0.363) | (0.365) | (0.377) | (0.358) |
| Liberal Arts | 0.594 | 0.595 | 0.636 | 0.577 |
|  | (0.392) | (0.393) | (0.395) | (0.390) |
| *Respondent’s Years at Current Institution* (*Reference: Less Than 1 Year)* | | | | |
| 1-5 years | 0.689 | 0.689* | 0.661 | 0.702* |
|  | (0.352) | (0.352) | (0.356) | (0.358) |
| 5-10 years | 1.027* | 1.027* | 1.028* | 1.039* |
|  | (0.411) | (0.412) | (0.417) | (0.414) |
| 10-20 years | 1.253** | 1.251** | 1.237** | 1.262** |
|  | (0.420) | (0.423) | (0.429) | (0.424) |
| More than 20 years | 0.617 | 0.616 | 0.585 | 0.617 |
|  | (0.460) | (0.459) | (0.478) | (0.465) |
| *Respondent’s Region* (*Reference: Northeast)* |  |  |  |  |
| Midwest | -0.414 | -0.413 | -0.420 | -0.410 |
|  | (0.310) | (0.311) | (0.314) | (0.310) |
| Southeast | 0.0274 | 0.0276 | 0.00994 | 0.0271 |
|  | (0.338) | (0.339) | (0.341) | (0.338) |
| West | -0.254 | -0.254 | -0.293 | -0.253 |
|  | (0.327) | (0.328) | (0.330) | (0.326) |
| *Respondent’s Location* (*Reference: Suburb & Rural)* | | | | |
| City | -0.140 | -0.143 | -0.132 | -0.139 |
|  | (0.281) | (0.281) | (0.282) | (0.281) |
| Town | -0.408 | -0.413 | -0.400 | -0.395 |
|  | (0.466) | (0.467) | (0.474) | (0.462) |
| *Couple Type* (*Reference: Heteronormative)* | | | | |
| Non-heteronormative | 0.149 | 0.151 | 0.160 | 0.150 |
|  | (0.634) | (0.639) | (0.639) | (0.625) |
| Other couple type | -1.462 | -1.516 | -1.000 | -1.382 |
|  | (0.821) | (0.953) | (0.907) | (0.826) |
| *Interaction Effects of Partner’s Attrition Reasons and Gender, Race, and Field* | | | | |
| Personal * Asian |  |  | 0.584 |  |
|  |  |  | (0.501) |  |
| Personal * Black |  |  | -0.640 |  |
|  |  |  | (1.032) |  |
| Personal * Hispanic |  |  | 0.425 |  |
|  |  |  | (0.793) |  |
| Personal * Not reported |  |  | -1.257 |  |
|  |  |  | (1.334) |  |
| Personal * Men |  | -0.00937 |  |  |
|  |  | (0.445) |  |  |
| Personal * Other genders |  | -0.276 |  |  |
|  |  | (1.904) |  |  |
| Personal * STEM |  |  |  | -0.162 |
|  |  |  |  | (0.530) |
| Personal * Medicine |  |  |  | -0.482 |
|  |  |  |  | (0.613) |
| Personal * Professional |  |  |  | -0.242 |
|  |  |  |  | (0.691) |
| Constant | 0.902 | 0.901 | 1.037 | 0.813 |
|  | (0.505) | (0.504) | (0.534) | (0.530) |
| N | 955 | 955 | 948 | 955 |

Robust standard errors in parentheses

*** p<0.001, ** p<0.01, * p<0.05

**Table S7. OLS Regression Results Showing Effects of Partner’s Attrition Reasons and Interaction Effects on Partner’s Salary**

|  | (1) | (2) | (3) | (4) |
| --- | --- | --- | --- | --- |
| Personal reasons | -0.677*** | -0.974*** | -0.788*** | -0.509* |
|  | (0.145) | (0.220) | (0.169) | (0.246) |
| *Partner’s Gender (Reference: Women)* |  |  |  |  |
| Men | 0.789*** | 0.614*** | 0.777*** | 0.805*** |
|  | (0.136) | (0.159) | (0.136) | (0.136) |
| Other genders | 0.652 | 0.641 | 0.544 | 0.630 |
|  | (0.677) | (0.754) | (0.694) | (0.681) |
| *Partner’s Race/Ethnicity (Reference: White)* |  |  |  |  |
| Asian | 0.0632 | 0.0525 | -0.122 | 0.0533 |
|  | (0.223) | (0.222) | (0.255) | (0.221) |
| Black | 0.147 | 0.131 | 0.0936 | 0.0897 |
|  | (0.285) | (0.288) | (0.336) | (0.278) |
| Hispanic | -0.490 | -0.461 | -0.439 | -0.512 |
|  | (0.305) | (0.306) | (0.370) | (0.301) |
| Not reported | -0.429 | -0.451 | -0.514 | -0.390 |
|  | (0.403) | (0.389) | (0.447) | (0.402) |
| Other Races | -0.233 | -0.254 | -0.582 | -0.246 |
|  | (0.540) | (0.548) | (0.703) | (0.535) |
| *Partner’s Field (Reference: Arts, Humanities, and Social Sciences)* | | | | |
| STEM | 0.840*** | 0.858*** | 0.847*** | 0.933*** |
|  | (0.176) | (0.175) | (0.176) | (0.207) |
| Medicine | 1.677*** | 1.701*** | 1.697*** | 1.574*** |
|  | (0.195) | (0.194) | (0.196) | (0.214) |
| Professional | 0.769*** | 0.786*** | 0.774*** | 1.068*** |
|  | (0.201) | (0.200) | (0.202) | (0.226) |
| *Partner’s Nativity (Reference: U.S.-born)* |  |  |  |  |
| Not U.S.-born | 0.00146 | 0.0107 | 0.0132 | 0.0146 |
|  | (0.159) | (0.159) | (0.160) | (0.157) |
| Nativity not reported | 1.092* | 1.063* | 1.074* | 1.058* |
|  | (0.463) | (0.456) | (0.478) | (0.447) |
| *Partner’s Years Since Degree (Reference: Up to 5 years)* | | | | |
| 6-10 years | 0.377 | 0.400 | 0.379 | 0.340 |
|  | (0.240) | (0.241) | (0.240) | (0.237) |
| 11-15 | 0.760** | 0.774** | 0.736** | 0.725** |
|  | (0.258) | (0.260) | (0.261) | (0.254) |
| 16-20 | 0.783** | 0.806** | 0.767** | 0.732** |
|  | (0.281) | (0.280) | (0.283) | (0.277) |
| 21-25 | 0.769* | 0.778* | 0.753* | 0.757* |
|  | (0.309) | (0.308) | (0.309) | (0.306) |
| 26-30 | 1.052** | 1.084*** | 1.027** | 1.001** |
|  | (0.328) | (0.327) | (0.330) | (0.330) |
| 31-35 | 1.166*** | 1.182*** | 1.137** | 1.102** |
|  | (0.345) | (0.342) | (0.347) | (0.342) |
| 36-40 | 0.684 | 0.712 | 0.692 | 0.669 |
|  | (0.449) | (0.456) | (0.456) | (0.445) |
| 41 or more years | 0.988* | 1.016* | 0.950* | 0.962* |
|  | (0.423) | (0.420) | (0.426) | (0.419) |
| Year unknown | 1.242*** | 1.292*** | 1.253*** | 1.106** |
|  | (0.367) | (0.376) | (0.370) | (0.379) |
| *Partner in same field* | -0.101 | -0.104 | -0.0946 | -0.128 |
|  | (0.142) | (0.142) | (0.142) | (0.142) |
| *No. of children* | 0.423 | 0.419 | 0.460 | 0.421 |
|  | (0.242) | (0.244) | (0.242) | (0.242) |
| *Carnegie Classification of Respondent’s Institution* (*Reference: Research 1)* | | | | |
| R2 | 0.0486 | 0.0407 | 0.0668 | 0.0982 |
|  | (0.229) | (0.225) | (0.228) | (0.229) |
| Master’s | -0.244 | -0.246 | -0.232 | -0.265 |
|  | (0.195) | (0.197) | (0.197) | (0.198) |
| Liberal Arts | -0.250 | -0.259 | -0.235 | -0.274 |
|  | (0.241) | (0.240) | (0.240) | (0.242) |
| *Respondent’s Years at Current Institution* (*Reference: Less Than 1 Year)* | | | | |
| 1-5 years | 0.322 | 0.350 | 0.290 | 0.244 |
|  | (0.320) | (0.319) | (0.321) | (0.327) |
| 5-10 | 0.567 | 0.610 | 0.523 | 0.495 |
|  | (0.334) | (0.333) | (0.336) | (0.342) |
| 10-20 years | 0.349 | 0.402 | 0.309 | 0.284 |
|  | (0.345) | (0.345) | (0.347) | (0.351) |
| More than 20 years | -0.157 | -0.131 | -0.209 | -0.204 |
|  | (0.373) | (0.373) | (0.377) | (0.377) |
| *Respondent’s Region* (*Reference: Northeast)* |  |  |  |  |
| Midwest | -0.290 | -0.271 | -0.293 | -0.318 |
|  | (0.188) | (0.188) | (0.190) | (0.185) |
| Southeast | -0.591** | -0.576** | -0.588** | -0.588** |
|  | (0.203) | (0.203) | (0.203) | (0.200) |
| West | 0.0702 | 0.0781 | 0.0598 | 0.0702 |
|  | (0.196) | (0.197) | (0.196) | (0.193) |
| *Respondent’s Location* (*Reference: Suburb & Rural)* | | | | |
| City | 0.135 | 0.140 | 0.137 | 0.105 |
|  | (0.181) | (0.181) | (0.181) | (0.179) |
| Town | -0.281 | -0.266 | -0.290 | -0.295 |
|  | (0.296) | (0.299) | (0.300) | (0.299) |
| *Couple Type* (*Reference: Heteronormative)* | | | | |
| Non-heteronormative | 0.400 | 0.446 | 0.442 | 0.447 |
|  | (0.289) | (0.297) | (0.294) | (0.290) |
| Other couple type | 0.205 | 0.236 | 0.338 | 0.284 |
|  | (0.799) | (0.812) | (0.834) | (0.789) |
| *Interaction Effects of Partner’s Attrition Reasons and Gender, Race, and Field* | | | | |
| Personal * Asian |  |  | 0.516 |  |
|  |  |  | (0.438) |  |
| Personal * Black |  |  | 0.158 |  |
|  |  |  | (0.566) |  |
| Personal * Hispanic |  |  | -0.172 |  |
|  |  |  | (0.640) |  |
| Personal * Not reported |  |  | 0.263 |  |
|  |  |  | (0.807) |  |
| Personal * Other races |  |  | 1.130 |  |
|  |  |  | (1.006) |  |
| Personal * Men |  | 0.562* |  |  |
|  |  | (0.284) |  |  |
| Personal * Other genders |  | -0.168 |  |  |
|  |  | (1.190) |  |  |
| Personal * STEM |  |  |  | -0.276 |
|  |  |  |  | (0.341) |
| Personal * Medicine |  |  |  | 0.666 |
|  |  |  |  | (0.423) |
| Personal * Professional |  |  |  | -1.056* |
|  |  |  |  | (0.412) |
| Constant | 2.828*** | 2.838*** | 2.891*** | 2.905*** |
|  | (0.422) | (0.421) | (0.422) | (0.426) |
| N | 740 | 740 | 740 | 740 |
| R-squared | 0.277 | 0.281 | 0.281 | 0.289 |

Robust standard errors in parentheses

*** p<0.001, ** p<0.01, * p<0.05
